# Lingual morphology of domesticated Asian small-clawed otters in Yogyakarta, Indonesia

**DOI:** 10.17221/62/2022-VETMED

**Published:** 2023-03-27

**Authors:** Angelina Kusuma Anjani, Golda Rani Saragih, Hevi Wihadmadyatami, Dwi Liliek Kusindarta

**Affiliations:** Department of Anatomy, Faculty of Veterinary Medicine, Gadjah Mada University, Yogyakarta, Indonesia

**Keywords:** *Aonyx cinereus*, electron and light microscopy, lingual glands, lingual papillae

## Abstract

This study aimed to observe the lingual morphology of the domesticated Asian small-clawed otter, *Aonyx cinereus* (*A. cinereus*), from Yogyakarta, Indonesia. Six domesticated *A. cinereus* adults were obtained from a local otter breeder in Yogyakarta, with no regard to sex. The animals were acclimated to the laboratory for one day, following this, the animals underwent macroscopy identification and scanning electron microscopy (SEM) and light microscopy (LM) analysis. Macroscopically, the tongue of domesticated *A. cinereus* is divided into three parts: the apex, corpus, and radix. The apex is the shortest part and can move freely. A median groove is bent along the corpus. Typically, the radix contains circumvallate papillae and the epiglottic valleculae. The SEM and LM observations revealed that the lingual morphology of *A. cinereus* consisted of two types of papillae: mechanical papillae (horny filiform, leaf-like filiform, bifid filiform, trifid filiform, elongated leaf-like filiform, triangular filiform and conical papillae) and gustatory papillae (fungiform and circumvallate papillae). The lingual glands consisted of Weber’s glands and von Ebner’s glands secreting acid and neutral mucins. Collagen fibres are found in the lamina propria and muscular layer. In conclusion, the papillae of the Asian short-clawed otter have the same structure as those of other *Mustelidae* family members.

The Asian small-clawed otter, or *Aonyx cinereus* (*A. cinereus)*, more commonly known to locals as *berang-berang*, is a semi-aquatic animal that is an excellent swimmer. This animal is found in Indonesia and in the south-eastern and eastern regions of Asia. *A. cinereus* belongs to the order Carnivora. *A. cinereus* has recently been adopted as a pet in Indonesia to replace dogs and cats due to its physical characteristics, which are similar to those of cats. This phenomenon increases the changes in the habitual behaviour of these animals and shifts the life pattern of *A. cinereus.* Originally a wild animal, it is becoming domesticated. Interestingly, this change in habitat encourages changes in the dietary patterns of *A. cinereus*. As the entrance organ to the digestive tract, the tongue of *A. cinereus* may be significantly impacted by changes in habitat (Williams 2019).

Recently, the large variation in the structures of the oral cavity, mainly in the lingua of the carnivora, has been related to adaptation to feeding methods, different types of food as well as the climate and habitat. Many studies concerning the dorsal structure of the lingual have been performed on carnivores, including golden Japanese weasels (Furubayashi et al. 1989), sea otters (Shimoda et al. 1996), bush dogs (Emura et al. 2000), panthers and Asian black bears (Emura et al. 2001), jaguars (Emura et al. 2013), black-backed jackals (Emura and Sugiyama 2014), fishing cats (Emura et al. 2014), sea lions (Erdogan et al. 2015), tigers (Emura et al. 2004), polar bears (Emura et al. 2017), cats (Emura 2018a) and leopards (Emura 2018b). These studies revealed variations in the morphology and distribution of the papillae on the dorsal lingual surfaces among the animal species. However, to the best of our knowledge, neither scanning electron microscopy (SEM) nor light microscopy (LM) studies of the tongue of the domesticated Asian short-clawed otter (*A. cinereus*) have been performed. Therefore, this study aimed to describe the morphological lingual surface of the domesticated *A. cinereus* in Yogyakarta, Indonesia.

## MATERIAL AND METHODS

### Animals

Six domesticated *A. cinereus* adults were obtained from local specialised otter breeders in Yogyakarta, Indonesia, with no regard to sex. The animals were obtained from a pet pond using the fish trap method and transported to the laboratory in an air-conditioned car equipped with *ad libitum* water. The animals were then run through a health check-up and acclimated for one day before being euthanised. Each animal was anaesthetised using 10 mg/kg body weight (b.w.) ketamine (Kepro, Maagdenburgstraat, The Netherlands) and 2 mg/kg b.w. xylazine (Interchemie, Metaalweg, The Netherlands), after which perfusion was performed. The species analysis was performed in the Laboratory of Animal Systems, Faculty of Biology, Gadjah Mada University.

### Conservation status

The *A. cinereus* used in this study do not appear on any conservation list in Indonesia and are not protected by the national laws of the Republic of Indonesia.

### Ethical approval

This research study was approved by the Ethics Clearance Committee of the Faculty of Veterinary Medicine, Gadjah Mada University (No. 0146/EC-FKH/Int/2019).

### Gross macroscopy analysis

The tongue of each animal was taken by first disjoining the articulatio temporomandibularis. The basis of the tongue was cut through the frenulum and the epiglottis. The tongue samples were rinsed with 0.9% NaCl (Nacalai Tesque, Kyoto, Japan). Macroscopic observations were conducted on the entire sample using a Canon EOS 7000 digital camera (Canon, Tokyo, Japan).

### Scanning electron microscopy

Four tongue samples were then fixed with 0.5% glutaraldehyde (Chem Cruz, Dallas, TX, USA), 1.5% formaldehyde, and 100 g HEPES (Chem Cruz, Dallas, TX, USA) in 100 ml phosphate buffered saline for 24–48 hours. The samples were then rinsed with 0.9% NaCl (Nacalai Tesque, Kyoto, Japan) and dehydrated using a graded series of ethanol, vacuum dried (1000 Vacuum System; Buehler, Stuttgart, Germany) and sputter coated (JEC-3000FC; Jeol, Tokyo, Japan) with platinum before being examined using Scanning Electron Microscopy (SEM) (JSM6510LA; Jeol, Tokyo, Japan) at an accelerating voltage of 15 kV.

### Measurements of the lingual papillae on the tongue of *A. cinereus*

To measure the length of the papillae, ImageJ software v1.53t was used (NIH, Maryland, USA). The SEM images were opened in the software; then, the measurement scale was equalised to the scale bar. Subsequently, a sample of papillae was selected, and a straight line was drawn from the tip to the base of the papilla to measure its length. To measure the width and diameter of the papilla, a straight line was drawn from end to end on the widest side of the papilla. The same steps were undertaken with a total sample of 20 papillae, measurements were performed a minimum of 3 times per papilla to minimise the error; then, the mean and standard deviation of each result was calculated. The measurements of each type of papillae are presented in Table 1.

**Table 1:** Distribution and measurements of the lingual papillae on the tongue of Aonyx cinereus

Type of papilla	Apex	Corpus	Radix	Length (μm) ± SD	Width (μm) ± SD
Horny filiform papilla	+	–	–	335.0 ± 3.7	130.0 ± 9.5
Leaf-like filiform papilla	+	–	–	280.7 ± 19.3	149.0 ± 13.2
Bifid filiform papilla	–	+	–	330.2 ± 25.2	150.0 ± 14.4
Trifid filiform papilla	–	+	–	356.2 ± 17.0	165.0 ± 15.4
Cornflower filiform papilla	–	+	–	406.2 ± 13.7	214.3 ± 18.1
Elongated leaf like papilla	–	+	–	546.2 ± 21.0	146.7 ± 18.0
Triangular filiform papilla	–	+	–	674.0 ± 34.8	311.0 ± 6.0
Short conical papilla	–	–	+	687.0 ± 8.3	279.2 ± 22.3
Long conical papilla	–	–	+	742.5 ± 19.3	366.4 ± 17.0
Fungiform papilla	+	+	+	460.9 ± 121.7	460.9 ± 121.7
Circumvallate papilla	–	–	+	237.8 ± 56.7	237.8 ± 56.7

The measurements were conducted for 20 samples for each papilla

SD = standard deviation

### Histological and histochemical staining

Two organ samples were fixed in a 4% paraformaldehyde solution (Nacalai Tesque, Kyoto, Japan) at a pH 7.4 for 48 h, and then trimmed accordingly to the apex, body and radix of the tongue. Subsequently, the tissue was sequentially processed by dehydration using graded ethanol (KgaA, Darmstadt, Germany); which was then cleared and embedded in a paraffin solution (Leica Biosystems, Wetzlar, Germany) and then cut, using a rotary microtome (Leica Biosystems, Wetzlar, Germany), with a thickness of 8 μm. The sample slides were then separately stained with histology staining using a haematoxylin and eosin (H&E) staining kit (Leica Biosystems, Wetzlar, Germany) and a Masson-Goldner trichrome staining kit (Bio-Optica, Milan, Italy), as well as histochemical staining using alcian blue (AB) staining at a pH 2.5 (Bio-Optica, Milan, Italy) and periodic acid-Schiff (PAS) (Bio-Optica, Milan, Italy).

### Light microscopy

Observations of the staining results of the samples were made using light microscopy (LM) with the microscope connected to Optilab software (Optilab, Yogyakarta, Indonesia). For the LM analysis, × 40 magnification was applied to all the samples.

## RESULTS

### Gross morphology of the tongue

**Figure 1:**
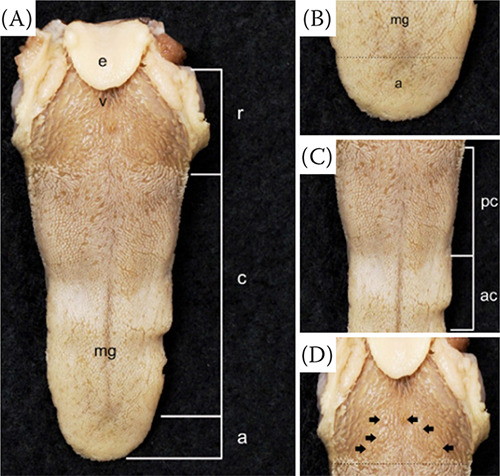
Gross macroscopy of the tongue of *Aonyx cinereus* (A) Macroscopic depiction of the tongue of *Aonyx cinereus*: (a) apex; (c) corpus; (r) radix; (e) epiglottis; (v) vallecula; (mg) median groove. (B) Macroscopic dorsal view of the apex of the tongue of *Aonyx cinereus*. (C) Macroscopic dorsal view of the corpus of the tongue of *Aonyx cinereus*: (ac) anterior corpus; (pc) posterior corpus. (D) Macroscopic dorsal view of the radix of the tongue of *Aonyx cinereus* (black arrow) circumvallate papilla

The tongue of *A. cinereus* is divided into three regions: the apex, corpus, and radix (Figure 1A). The tongue has four surfaces: one dorsal, one ventral, and two lateral ones. There are two boundaries between the apex and corpus: the frenulum and the median groove. The frenulum connects the ventral surface of the tongue with the mandibular and the initial tip of the median groove. The border between the corpus and the radix is the end of the median groove. The apex is the very front region of the tongue. The apex is the shortest part of the tongue, but it can move freely to help with food intake (Figure 1B). The corpus is the longest area on the tongue of *A. cinereus*, and it is divided into two parts: the anterior and posterior corpora. The median groove of the corpus extends from the anterior part of the corpus to the posterior part (Figure 1C). The radix is the rearmost part of the tongue and is connected to the larynx. The radix contains six circumvallate papillae that lead to the larynx (Figure 1D).

### Scanning electron microscopy

#### APEX

**Figure 2:**
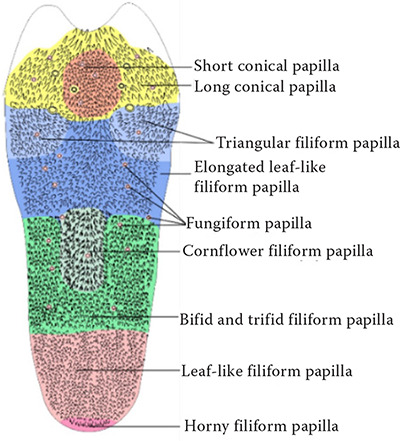
The schematic illustration of the papillae distribution on the tongue of *Aonyx cinereus*

**Figure 3:**
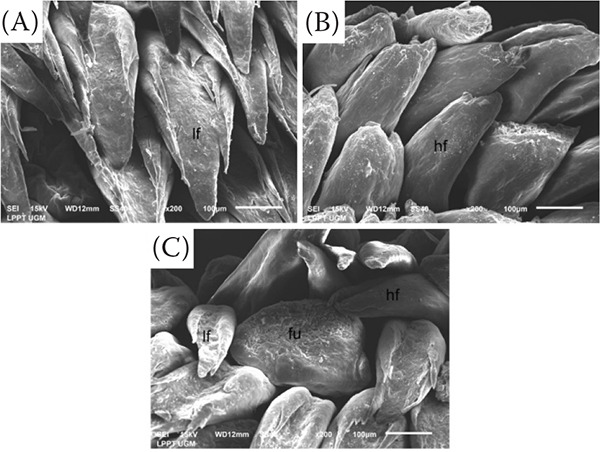
Scanning electron microscopy (SEM) images of papillae on the apex region of the tongue of *Aonyx cinereus* (A) Leaf-like filiform papillae that occupy most parts of the apex: (lf) leaf-like filiform papilla. (B) Horny filiform papilla that occupies a small portion on the tip of the tongue: (hf) horny filiform papilla. (C) Fungiform papilla between the filiform papillae: (fu) fungiform papilla

Three types of papillae were found on the dorsal apex surface of the tongue: leaf-like filiform, horny filiform and fungiform papillae (Figure 2, Table 1). Leaf-like filiform papillae are found on almost all the surfaces of the apex. Leaf-like filiform papillae are long, have sharp edges and 5–6 processes that lead to the caudal area of the tongue (Figure 3A). Horny filiform papillae are found in a small area at the apex tip. Horny filiform papillae have a long blunt tip that leads to the cranial of the apex (Figure 3B). The fungiform papillae spread between the leaf-like and horny filiform papillae. Fungiform papillae have smooth and round surfaces (Figure 3C).

#### CORPUS

**Figure 4:**
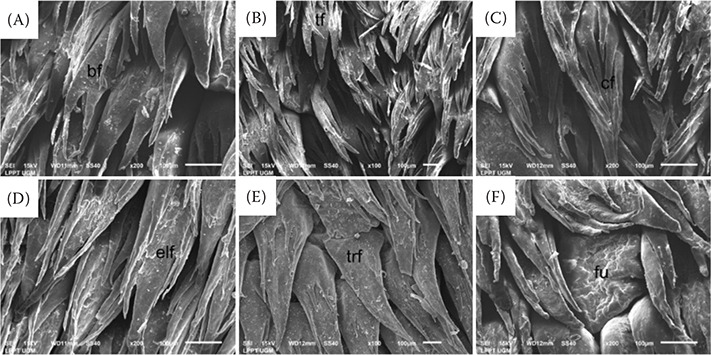
Scanning electron microscopy (SEM) images of papilla on the corpus region of the tongue of *Aonyx cinereus* (A) Bifid filiform papilla that occupies anterior corpus alongside trifid filiform papilla; (bf) bifid filiform papilla. (B) Trifid filiform papilla that occupies anterior corpus alongside bifid filiform papilla; (tf) trifid filiform papilla. (C) Cornflower filiform papilla that occupies posterior section on the anterior corpus; (cf) cornflower filiform. (D) Elongated leaf-like filiform papilla occupying posterior corpus; (elf) elongated leaf-like filiform. (E) Triangular filiform papilla occupying the latero-posterior section on posterior corpus; (trf) triangular filiform papilla. (F) Fungiform papilla that scatters between filiform papilla on the corpus of the tongue; (fu) fungiform papilla

The papillae found in the corpus are divided into two regions: the anterior and posterior corpora. In the anterior corpus, there are bifid filiform, trifid filiform and cornflower filiform papillae; in the posterior corpus, there are elongated leaf-like and triangular papillae (Figure 2, Table 1). The bifid and trifid filiform papillae are adjacent to one another. Bifid filiform papillae have a special characteristic: the posterior tip has two processes that lead to the caudal area and form the bifid in this papilla (Figure 4A). The trifid filiform papilla also has a special characteristic: three processes at the edges lead to the caudal area, forming a trifid (Figure 4B). The cornflower filiform papillae are located in the posteromedial part of the anterior corpus. Cornflower filiform papillae have many processes that lead to the caudal area. These papillae have a shape that is similar to the leaf-like filiform papilla, but their process in the lateral section folds to the centre, creating a more prominent side, as if there is a gap in the middle of the papilla (Figure 4C). The elongated filiform papillae have a long, sharp tip, and there are many processes around them that lead to the caudal area (Figure 4D). The triangular filiform papillae are located in the posterolateral part of the posterior corpus. This filiform papilla has sharp edges and a triangular shape. It delimits the corpus and radix areas on the right and left sides of the tongue (Figure 4E). Fungiform papillae are also found in the corpus, scattered among the filiform papillae (Figure 4F). The median groove is in the middle of the corpus; it is a long groove that seems to divide the tongue into two parts (Figure 1A).

#### RADIX

**Figure 5:**
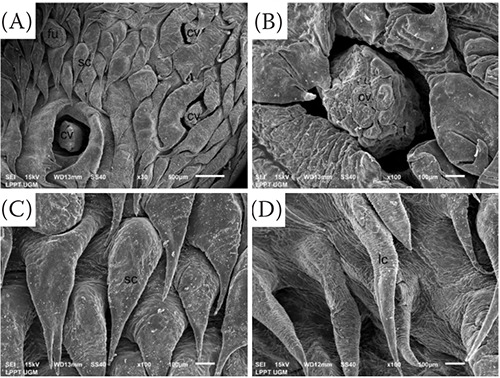
Scanning electron microscopy (SEM) images of papillae on the radix region of the tongue of *Aonyx cinereus* (A) half section of the radix regio; (cv) circumvallate papilla; (sc) short conical papilla; (fu) fungiform papilla. (B) higher magnification of the circumvallate papilla. (C) short conical papilla occupying the median section of the radix; (sc) short conical papilla. (D) long conical papilla occupying the lateral section of the radix; (lc) long conical papilla

In the radix, the tongue consists of several types of papillae: short conical, long conical and circumvallate with fungiform papillae (Figure 5A, Figure 2, Table 1). The short and long conical papillae are spread over almost all the radix surfaces. The short conical papilla leads to the caudal area and has a flat tip. The size is smaller and the base is rounder than that of the long conical papilla (Figure 5C). This papilla is located in the medial radix. The long conical papilla leads to the caudomedial area, and the tip is flat. Its size is longer than that of the short conical papilla (Figure 5D). This papilla is located lateral to the radix.

The circumvallate papillae are located at the base of the tongue, close to the larynx. There are six circumvallate papillae that form a “V”, with the angle leading to the larynx.

The shape of a circumvallate papilla is similar to that of a fungiform papilla; its surface is smooth and has no processes. This type of papilla has an outer wall and a groove that follows the shape of the papilla (Figure 5B). The fungiform papilla is spread between the short and long conical papillae.

### Light microscopy – Haematoxylin and eosin staining

#### APEX

**Figure 6:**
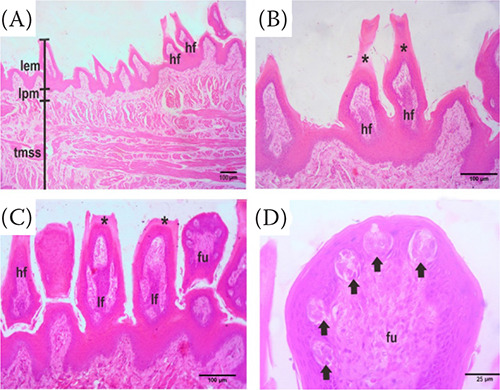
Photomicrograph of the *Aonyx cinereus* tongue apex region, with haematoxylin and eosin staining (A) Apex region histologically divided into three layers: (lem) lamina epithelialis mucosae; (lpm) lamina propria mucosae; and (tmss) textus muscularis striatus syncytialis. The tip of the apex is dominated by horny filiform (hf) papillae. (B) Higher magnification of horny filiform (hf) with thick keratinisation (*) which are elongated and form a concave in the dorsal end. (C) Transition part from the anterior tip showing horny filiform (hf) and leaf-like filiform (lf) with elliptical epithelial layer covered by thick keratinisation forming lateral processes. Fungiform (fu) papilla is located among the filiform papillae. (D) Higher magnification of a dome-shaped fungiform (fu) papilla, characterised with several taste buds (arrow) in the inner epithelial layer and covered by a thin keratin layer

There are two distinct forms of filiform papillae at the apex of *A. cinereus*: horny filiform and leaf-like filiform (Figure 6). The filiform horny papillae dominate the anterior end region, with thick keratinised formations extending upward and forming a depression at the tip (Figure 6B). The leaf-like filiform papillae have wider bodies than the horny filiform papillae, with thick keratinisation-forming processes at their lateral ends (Figure 6C). Moreover, the apex has fungiform papillae with a rounded tip resembling a dome and five to seven taste buds visible on the inner layer of the epithelium (Figure 6D).

#### CORPUS

**Figure 7:**
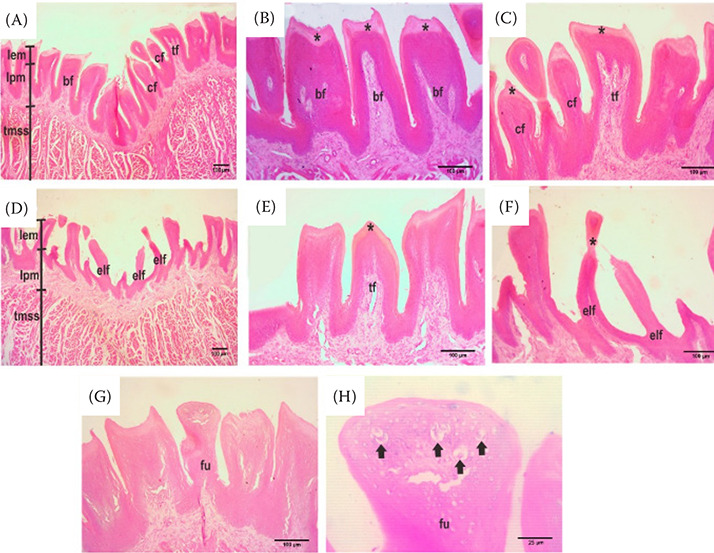
Photomicrograph of the Asian small-clawed otter’s tongue corpus region, with haematoxylin and eosin (H&E) staining (A) Anterior corpus histologically divided by three layers: (lem) lamina epithelialis mucosae; (lpm) lamina propria mucosae; and (tmss) textus muscularis striatus syncytialis. The surface of the mucous layers forming papillae named cornflower filiform (cf), bifid filiform (bf), and trifid filiform (tf). (B) Higher magnification of bifid filiform papilla (bf) with elongated lamina propria branched into two. Thick keratin layer (*) forming pointed lateral tips. (C) Higher magnification of trifid filiform papilla (tf) with three branches of elongated lamina propria, covered by a thick keratin layer (*). Cornflower papillae (cf) is covered by a bud-like keratin layer (*). (D) Posterior corpus histologically divided by three layers: (lem) lamina epithelialis mucosae; (lpm) lamina propria mucosae; and (tmss) textus muscularis striatus syncytialis. There are elongated leaf-like filiform (elf) papillae in the medial and triangular filiform (tf) papillae in the lateral part. (E) Higher magnification of triangular filiform papilla (tf) forming a pointed shape in the upper one-third part, covered by thick keratinisation (*). (F) Higher magnification of elongated leaf-like filiform (elf) papilla that has a lean elongated shape with thick keratinisation (*). (G) Fungiform papillae (fu) located among the filiform papillae. (H) Higher magnification of fungiform papillae (fu), equipped with several taste buds (arrow)

The anterior and lateral ends of the anterior lingual corpus of the tongue samples are occupied by bifid and trifid filiform papillae (Figure 7A–C). Both subtypes are located next to each other and have morphologically similar formations. They are similar in that they have an epithelial lamina that resembles a rectangular building with a flat or slightly concave tip and a thick layer of keratin that forms short horns/processes on the lateral side of the papilla. The difference between them is that the lamina propria in the bifid filiform has either only a single tip or branches into two tips; the trifid filiform has a broader lamina propria that branches into three tips. The medial part contains filiform cornflower papillae with elliptical epithelial laminas and is covered by a thick layer of keratin, with a bud-like formation at the ends (Figure 7C).

The posterior corpus of the tongue samples is filled with elongated leaf-like filiform papillae and triangular filiform papillae (Figure 7D,E). The leaf-like filiform papillae are slender and elongated, with thick keratinisation similar to the shape of the epithelial lamina at their dorsal ends (Figure 7F). The triangular filiform papillae are recognised by their conical shape at one-third of their tips, which points dorsally and has thick keratinisation (Figure 7E). The fungiform papillae in the corpus are similar to those in the apex, with a dome-like shape and several taste buds (Figure 7G,H).

#### RADIX

**Figure 8:**
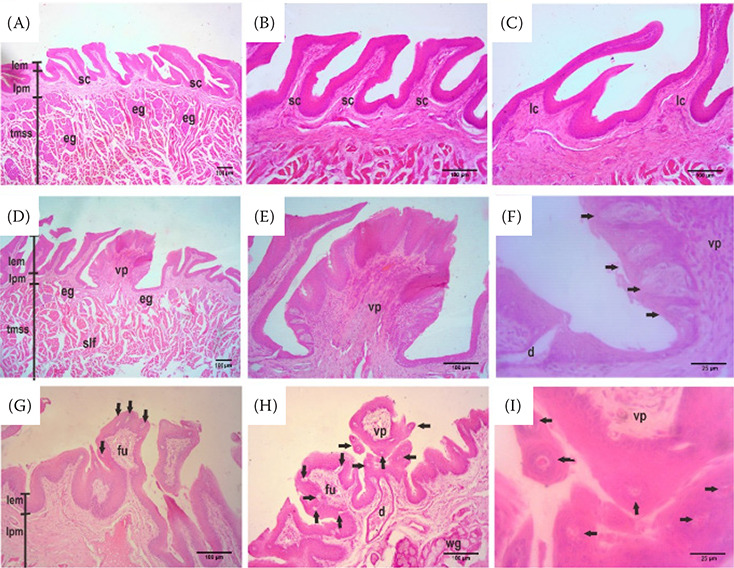
Photomicrograph of the Asian small-clawed otter’s tongue radix region, with haematoxylin and eosin (H&E) staining (A) Three layers of radix: (lem) lamina epithelialis mucosae; (lpm) lamina propria mucosae; and (tmss) textus muscularis striatus syncytialis. Short conical papillae (sc) is located in the medial part of radix with von Ebner’s gland (eg) underneath. (B) Higher magnification of short conical papillae (sc). (C) Higher magnification of long conical papillae (lc) that are located in the lateral part of radix. (D) Circumvallate papillae (vp) in the anterior radix, surrounded by a groove and showing von Ebner’s gland (eg) underneath. (E) Higher magnification of circumvallate papillae (vp) that have round shape with the surface lamina propria mucosae layer forming processes. (F) Higher magnification of the ventral part of circumvallate papillae (vp), which are equipped by taste buds (arrow) in the ventrolateral part and lingual gland’s duct (d) in the base of the groove. (G) Fungiform papillae (fu) in the posterior radix with taste buds in the dorsal and lateral surface. (H) Another form of fungiform papillae (fu) and circumvallate papillae (vp) in the posterior radix. Both have numerous taste buds in the epithelial layer (arrow). Underneath the circumvallate papillae, Weber’s gland (wg) is equipped with a duct (d). (I) Higher magnification of the ventral part of circumvallate papillae (vp) showing taste buds (arrow) in the ventrolateral surface

The anterior radix of the samples have conical papillae, with short conical papillae in the medial part of the tongue (Figure 8B) and longer ones, known as long conical papillae, in the lateral part (Figure 8C). Short conical papillae are formed from elevatio-ns of the lamina propria with a flattened epithelial plate at the ends. In contrast, the long conical papillae are elliptical, extend upward with a rounded tip. The posterior radix contains the gustatory papillae and the lamina epithelialis, which have irregular shapes and numerous taste buds scattered on its surface.

The circumvallate papillae of *A. cinereus* vary in shape. However, they all have things in common, such as the presence of a groove around the papilla, an abundance of taste buds on the papilla’s lateral and ventral epithelial sides and the presence of a lingual gland at the base of the circumvallate papilla. The circumvallate papillae that are located anteriorly have a rounded structure (Figure 8E), while those located posteriorly vary. One of these papillae is in the form of an almost separate building with a protrusion of the oval epithelial lamina that contains taste buds (Figure 8H,I). The fungiform papillae located on the posterior radix have different shapes without any observable characteristic features, apart from the presence of several taste buds on the dorsal and lateral epithelial lamina (Figure 8G,H). The keratinised layer was not observed in the papillae at the radix of the *A. cinereus* tongue samples.

### Lingual glands

**Figure 9:**
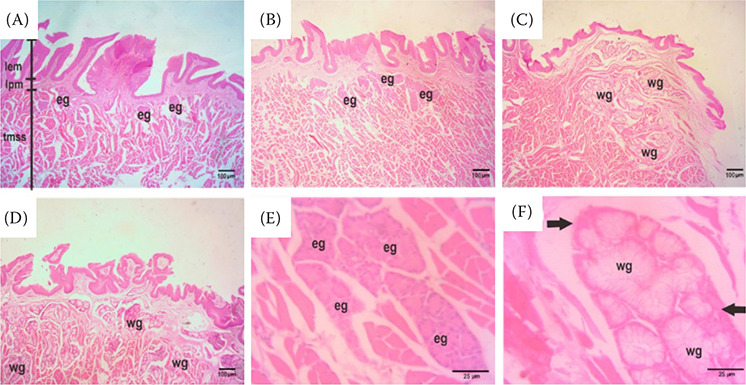
Location of the lingual glands in the radix region of Asian small-clawed otter’s tongue with haematoxylin and eosin (H&E) staining (A) Von Ebner’s gland found in the anterior part of the radix, located in upper layer of textus muscularis striatus syncytialis (tmss) under the circumvallate papillae: (lem) lamina epithelialis mucosae; (lpm) lamina propria mucosae. (B) Von Ebner’s gland (eg) in the medial of the middle part of radix. (C) Weber’s gland (wg) in the lateral of the middle part of radix. (D) Weber’s gland in both medial and lateral of posterior part of the radix. (E) Higher magnification of von Ebner’s gland (eg), showing the structure of the serous gland. (F) Higher magnification of Weber’s gland (wg), showing the structure of seromucous gland that is dominated by mucous cells and a small number of serous cells (arrow)

The lingual glands in *A. cinereus* can be divided into Weber’s glands and von Ebner’s glands (Figure 9). These glands have different positions, structures and biochemistry. Weber’s glands are lateral seromucous glands, with a predominance of mucous cells located in the lamina propria and muscularis. The medial gland is a serous gland, known as von Ebner’s gland; it is primarily located at the base of the circumvallate papillae or more deeply located between the muscles of the tongue. Weber’s glands contain only a few serous cells; some of them form demilune caps, while the rest are separate clusters between the mucus cells. The serous cells had a spherical nucleus with acidophilic cytoplasm on the H&E staining (Figure 9E). Furthermore, Weber’s glands consist of mucous cells and serous cells. Mucous cells had characteristic flattened nuclei located on the basement membrane and cytoplasm and were stained pale with the H&E staining (Figure 9F).

**Figure 10:**
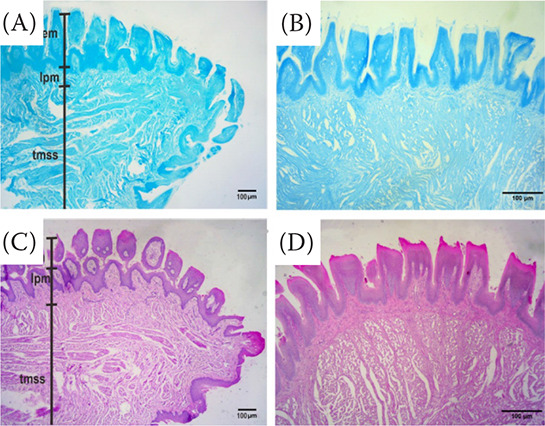
Photomicrograph of the apex and corpus region of Asian small-clawed otter’s tongue with alcian blue (AB) and periodic acid-Schiff (PAS) staining, showing no positive reaction to the lingual gland’s secretion (A) Apex region, AB stain: (lem) lamina epithelialis mucosae; (lpm) lamina propria mucosae; and (tmss) textus muscularis striatus syncytialis. (B) Corpus region, AB stain. (C) Apex region, PAS stain: (lem) lamina epithelialis mucosae; (lpm) lamina propria mucosae; and (tmss) textus muscularis striatus syncytialis.(D) Corpus region, PAS stain

The lingual glands in *A. cinereus* were observed in the lamina propria and textus muscularis striatus syncytial from the radix lingua; no glandular formations or histochemical reactions from mucin secretion were observed in the apex or corpus area (Figure 10). There are variations in the glands in the anterior radix and the glands in the posterior radix. The anterior radix contains some serous glands (von Ebner’s glands) in the upper muscularis layer from around the base of the circumvallate papillae. As one looks more posteriorly, the organ appears to contain more seromucous glands (Weber’s glands). The serous glands are seen only on the medial side of the tongue, whereas the seromucous glands are initially located laterally until they replace the serous glands on the medial side (Figure 10).

### Alcian blue and periodic acid-Schiff

**Figure 11:**
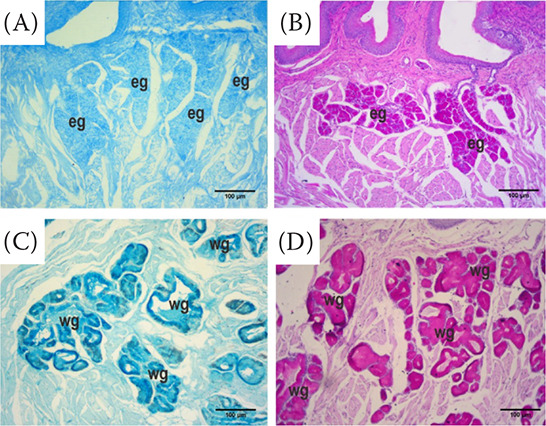
Photomicrograph of the radix region of Asian small-clawed otter’s tongue with alcian blue (AB) and periodic acid-Schiff (PAS) staining (A) Medial part of radix shows von Ebner gland (eg) with a weak positive (+) reaction under a gustatory papillae, AB stain. (B) Von Ebner’s gland (eg) with a moderate positive (++) reaction, PAS stain. (C) Lateral part of radix shows Weber’s glands (wg) with moderate positive (++) reaction which located in the textus muscularis striatus syncytialis, AB stain. (D) Weber’s gland with a strong positive (+++) reaction, PAS stain

The composition and intensity of the mucin produced by the lingual glands differ. Histochemical staining with alcian blue (AB) and periodic acid-Schiff (PAS) can illuminate this. The AB staining of von Ebner’s glands showed a weak positive (+) result (Figure 11A). Weber’s glands were dark blue, showing a moderate positive reaction (++) (Figure 11C). A positive reaction for AB staining at pH 2.5 indicates the presence acid mucin (sialomucin and sulfomucin) in both glands.

The PAS staining showed positive results in all the glands found in the radix of the tongue. The von Ebner’s glands were coloured purple, which showed a moderate positive reaction (++) (Figure 11B). Weber’s glands were stained magenta, showing a strong positive reaction (+++) (Figure 11D). A stronger positive reaction to PAS staining indicates a more neutral mucin produced by the glands.

### Masson-Goldner trichrome staining

**Figure 12:**
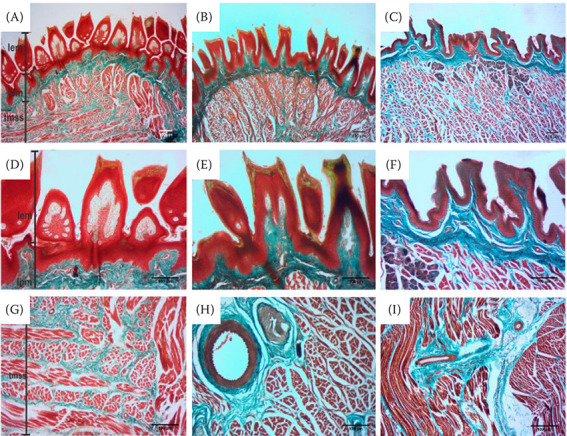
Photomicrograph of Asian small-clawed otter’s tongue with Masson’s Trichrome staining (A) Apex region of the tongue, showing various shapes of epithelial tissue in lamina epithelialis mucosae (lem), dense collagen fibres in lamina propria mucosae (lpm), and looser collagen fibres in textus muscularis striatus syncytialis (tmss). Corpus (B) and radix (C) part shows the same as apex. (D) Higher magnification of mucous layer of the lingual apex including the lamina epithelialis mucosae (lem) and lamina propria mucosae (lpm). (E) Mucous layer of the lingual corpus. (F) Mucous layer of the lingual radix. (G) Higher magnification of textus muscularis striatus syncytialis (tmss) layer in the lingual apex, showing loose collagen fibres (stained in blue) between the longitudinal and transverse muscle fibres (stained in red). (H) Muscular layer of the lingual corpus. (I) Muscular layer of the lingual radix

The collagen present in the connective tissue produces a blue-green colour in the Masson-Goldner trichrome staining. Collagen is abundant in the lamina propria under the tongue’s papillae and the textus muscularis striatus syncytialis tissue between the muscle fibres with a lower intensity (Figure 12). In the *A. cinereus* tongue samples, the collagen intensity from the apex to the radix did not change.

## DISCUSSION

In mammals, the feeding behaviour is an important factor determining the vertebrates’ successful adaptation to their environment. Mammalian feeding has been the subject of intense study from comparative, functional, and biomechanical perspectives (Williams 2019). From the biomechanical perspective, the tongue – along with other organs in and near the oral cavity – plays a major role in the feeding activity (Iwasaki 2002). According to the process model, the feeding sequence requires the tongue to bring food into the oral cavity. If the food is palatable, it is transported to the post-canine area. If not palatable, it is expelled from the oral cavity. When food reaches a swallowable state, it is transported through the fauces to the vallecula and piriform fossae, where bolus aggregation occurs. Upon the formation of an adequate bolus, swallowing is triggered and the food is forced into the oesophagus (Williams 2019).

The tongue morphology, in its macroscopic structure (tongue papillae types, shape, function, and distribution) in vertebrates, varies to fit their diets (i.e., whether they are carnivores, omnivores, or herbivores) as well as their environments and habitats, which may have played a partial role in the evolution of the tongue’s structure (Iwasaki et al. 2019). Such an adaptation based on the diet, environment, or habitat has been described in various studies, especially in mammals, such as goats (Mahdy et al. 2021), pumas (Erdogan et al. 2018), sugar gliders (Damia et al. 2021), and several other carnivores, including polar bears, marsupials, and bats (Emura et al. 2017; Gozdziewska-Harlajczuk et al. 2020; Gunawan et al. 2020). In a study, the tongue morphology revealed a relationship between changes in the habitat and changes in the appearance of the tongue; the transition from a freshwater (moist or wet) environment to a terrestrial (dry) environment resulted in the keratinisation of the lingual epithelium (Iwasaki 2002).

Recently, in Indonesia, the Asian short-clawed otter, *A. cinereus*, has been widely used as a pet. Since *A. cinereus* became a pet, many changes have occurred, primarily related to the shape and type of food eaten by this animal. In the wild, *A. cinereus* usually consumes fresh fish, but when it is adopted as a pet, it also eats other foods, such as meat, cat food, and enrichment food (crayfish, mealworms, snails, and crickets). This may contribute to structural changes in the types of papillae found on the dorsal surface of the tongue. Previous SEM studies have been conducted on a variety of carnivores; the present study reported numerous variations in the type of lingual papillae. The study explored the papillae and lingual gland structures using the tongues of domesticated *A. cinereus* derived from a local breeder in Yogyakarta, Indonesia.

From the macroscopic observations, the tongue formations in *A. cinereus* consisted of three parts: the apex, corpus and radix (Figure 1A). The apex is concise and can move freely. The corpus is identical to the medial groove, which bends along with the corpus. The radix has six circumvallate papillae, the vallecula epiglottis and the epiglottis (Figure 1). The morphological description of the tongue of *A. cinereus* is the same as the morphological differentiation of the lingua from other orders of carnivores.

The scanning electron microscopy of the dorsal surface of *A. cinereus* revealed two types of papillae based on their function: mechanical papillae and gustatory papillae. Mechanical papillae consist of different types of filiform papillae (horny filiform, leaf-like filiform, bifid filiform, trifid filiform, elongated leaf-like filiform and triangular filiform papillae) and conical papillae. The gustatory papillae are divided into fungiform and circumvallate papillae. Fungiform papillae were found scattered along the dorsal surface of the tongue from the apex to the radix. Foliate papillae were not found in this study. Instead, the function and position of the foliate papillae are replaced by large numbers of conical papillae. Unlike foliate papillae, conical papillae do not have taste buds.

The observations of the apex revealed three types of filiform papillae. Interestingly, on the apex tip, we found horny filiform papillae; on another area of the apex, we found leaf-like filiform papillae and fungiform papillae (Figure 3). Recent data have shown that horny filiform papillae appear in newborn sea otters (Shimoda et al. 1996). According to our observations, horny filiform appears in adult otters. Thus, we can postulate that these papillae are not just a stage in the papillae’s development, but also the type of papillae found in adult otters. These horny filiform papillae have an essential mechanical function during suckling. It is possible that these structures on the tip of the apex help adult otters consume food.

On the corpus, the SEM observations revealed numerous filiform papillae in the anterior and posterior corpora. In the anterior corpus, there were bifid filiform, trifid filiform, and cornflower filiform papillae. In the posterior corpus, there were elongated leaf-like and triangular papillae (Figure 4). Furthermore, the corpus was especially marked by regional variations in the size and morphology of the filiform papillae, which structurally consist of a large main papilla and some secondary papillae; thus, they have a bifid, trifid, elongated leaf-like and mountain-like shape – or, what we refer to as triangular filiform papillae. Our finding is consistent with the observations previously reported on the mid-area lingua of the fishing cat (Emura et al. 2014) and the common raccoon (Miyawaki et al. 2010).

In the radix, we found six circumvallate papillae, with a “V” formation pointing to the larynx (Figure 1 and Figure 5). The SEM and LM observations showed that the circumvallate papillae have a deep groove and pad in their surroundings (Figure 5); this groove serves to improve the accessibility of food to the taste buds in this type of papilla.

The formation of circumvallate papillae found in this study is in line with the results reported in a study on jaguars (Emura et al. 2013), a study on *A. cinereus* in Japan (Emura and Sugiyama 2016), a study on English horses (Can et al. 2016), a study on *Bubalus bubalis* (El-Bakary and Abumandour 2017) and a study on bats (Gunawan et al. 2020). The number of circumvallate papillae is slightly lower in *A. cinereus* than in leopards, which belong to the same order as *A. cinereus* (leopards have seven circumvallate papillae) (Emura 2018b). Several studies have reported variations in the numbers of circumvallate papillae; for example, four circumvallate papillae were observed in tigers (Emura et al. 2004). In contrast, five circumvallate papillae were observed in bush dogs (Emura et al. 2000). There were ten circumvallate papillae in panthers and seven or eight in Asian black bears (Emura et al. 2001). Five to nine circumvallate papillae were observed in lions (Erdogan et al. 2015). Seven circumvallate papillae were observed in jaguars (Emura et al. 2013). This variation is influenced by the sensitivity of the taste receptors (Wolczuk 2014).

In the radix, we observed long conical and short conical papillae positioned laterally and medially to the circumvallate papillae (Figure 5). These papillae have also been observed on the lingual radix of other carnivores, such as pumas (Erdogan et al. 2018), wolves (Haligur et al. 2019), Japanese black bears (Inatomi and Kobayashi 1999) and common raccoons (Miyawaki et al. 2010). The abundance of mechanical papillae indicates that these papillae play an important role in mastication and deglutition (Inatomi and Kobayashi 1999). The thick keratin layer found in the outermost layer of the mechanical papilla protects against the mechanical stimuli from the food consumed (Gozdziewska-Harlajczuk et al. 2016).

Finally, similar to the apex and corpus, we observed fungiform papillae scattered along the lingual radix of *A. cinereus* (Figure 5). Animals of the *Mustelidae* family possess multiple variations in fungiform papillae, such as the Japanese weasel, which has fungiform papillae with increasing size and amount at the apex (Furubayashi et al. 1989), in contrast to ferrets, whose fungiform papillae are more abundant in the radix lingua. Although most fungiform papillae are either spherical or bud-like, some species have other shapes, such as the square-shaped papilla in the ferret (Takemura et al. 2009). The Asian small-clawed otter (*A. cinereus*) has fungiform papillae with 5–7 taste buds on the LM that are club-shaped from the apex to the anterior radix and vary in shape on the posterior radix. This resembles the fungiform papillae on the tongue of the sea otter (Shimoda et al. 1996). Data from previous studies have shown that the type of feed consumed by an animal affects the number of taste buds on its tongue (Wolczuk 2014).

Vertebrates have salivary glands, whose main purpose is to maintain mucosal moisture, lubricate food and initiate chemical substances in food processing in the oral cavity. Salivary glands contain major and minor glands. Minor salivary glands are found in the oral submucosa of animals, one of which is the lingual glands. The lingual glands in *A. cinereus* were observed only in the radix, as is the case in other animals in the *Mustelidae* family, such as ferrets (Takemura et al. 2009). Two types of lingual glands can be found: von Ebner’s glands, which are serous glands, and Weber’s glands, which are seromucous glands. Mucous produced from the seromucous lingual glands describe as a slippery substrate to facilitate the swallowing of dry food and support tongue movement; serous gland secretion plays a role in tasting because it is located around the main gustatory papilla, the circumvallate papilla (Haddo and Yasear 2018).

The distribution of the location of the lingual glands in *A. cinereus* is the same as in primates. In the anterior radix, there are serous glands (von Ebner’s glands); in the posterior radix, there are mucous dominant glands (Weber’s glands); in between, von Ebner’s glands are located medially and Weber’s glands are located laterally. The dispersal pattern is considered to support the ingestion of food boluses and is associated with the protection of the oral cavity (Yoshimura et al. 2019). The histochemical staining showed that the mucin secretions from both types of lingual glands were acid and neutral mucin with a higher intensity in Weber’s glands than in von Ebner’s glands. Kadhim et al. (2013) stated that the neutral mucin secreted by the lingual glands acts as a feed lubricant for the swallowing process and maintains hydration by providing a hydrophilic environment. Acid mucin aids in oral protection because it can modulate the oral calcium channel activity.

The connective tissue is the main structure that links tissues to form organs. The extracellular matrix (ECM) is a major component of the connective tissue, and collagen is a critical element of the ECM (Mescher 2016). The dense connective tissue dominated by collagen (stained in darker blue using Masson-Goldner trichrome) shows the density of collagen seen in the lamina propria, while irregular, loose connective tissue can be found in the muscular layer between the muscle fibres and other tissues. Like the human tongue, the collagen fibres in the papillary layer (the lamina propria on the papilla) in the *A. cinereus* tongue samples were markedly thin and loose. In the deeper reticular layer, collagen fibres are arranged densely and tightly (Walther et al. 2019).

Variations in the collagen intensity between parts of the tongue in the *A. cinereus* samples were not clearly observed in this study. This is presumably due to the size of the tongue, which is large and thick, so a strong collagen layer is required from the apex to the radix to support the main building of the tongue, which is composed of muscles.

In conclusion, structurally, the papillae of the Asian short-clawed otter (*A. cinereus*) from Yogyakarta, Indonesia, consist of numerous types of filiform papillae, including horny filiform papillae and gustatory papillae. Lingual glands secrete neutral and acid mucins, which aid in the processing and swallowing of food. Thus, both the lingual morphological structure and the lingual glands of domestic *A. cinereus* are similar to those of other members of the *Mustelidae* family.
